# Postmortem Methanol and Formic Acid Levels and Their Pathological Correlates: Diagnostic Implications from an Autopsy Study

**DOI:** 10.3390/diagnostics16020233

**Published:** 2026-01-11

**Authors:** Filiz Ekim Çevik, Aytül Bugra, Hüseyin Cagri Sahin, Muhammed Oduncu, Sümeyye Demirdöven Özbakir, Hızır Asliyüksek

**Affiliations:** 1 Department of Medical Sciences, Institute of Forensic Sciences and Legal Medicine, Istanbul University-Cerrahpasa, Istanbul 34500, Türkiye; 2 Council of Forensic Medicine, Istanbul 34186, Türkiye

**Keywords:** methanol poisoning, formic acid, forensic medicine, postmortem diagnosis, autopsy, toxicology

## Abstract

**Background:** Methanol poisoning remains a major cause of fatal toxic exposures worldwide, yet the diagnostic value of postmortem methanol and formic acid levels in relation to organ-specific pathology is not fully understood. This study aimed to provide a comprehensive forensic and diagnostic evaluation of fatal methanol intoxications using multiple biochemical and pathological parameters. **Methods:** A total of 138 autopsy-confirmed methanol poisoning cases were retrospectively analyzed. Quantitative methanol and formic acid levels were measured in blood and vitreous humor. Autopsy reports, demographic characteristics, and histopathological findings in major organs were systematically reviewed. The presence of ethanol and other substances, including stimulants and narcotic drugs, was also recorded. **Results:** Blood methanol concentrations averaged 142.47 ± 139.20 mg/dL (range: 0–595), and formic acid levels averaged 258.62 ± 197.89 mg/dL (range: 0–618). Vitreous humor concentrations showed comparable distributions. Common pathological findings included cerebral edema, putaminal discoloration or necrosis, myocardial ischemia, hepatic steatosis, pulmonary edema, and acute pancreatitis. Ethanol or other substances were detected in several cases, with stimulants or narcotic drugs present in 10.4% (*n* = 13). Importantly, the combined interpretation of postmortem biochemical markers and organ pathology allowed clearer differentiation of methanol-related injury patterns compared with prior reports. **Conclusions:** Methanol intoxication produces variable but characteristic biochemical and pathological profiles. Integrating toxicological markers with organ-specific pathology enhances the diagnostic accuracy of postmortem evaluations and supports more reliable identification of methanol-related deaths.

## 1. Introduction

Outbreaks of methanol poisoning have been documented in the medical literature worldwide, underscoring a significant global public health concern. Methanol (CH_3_OH), also known as methyl alcohol or wood alcohol, is widely used as a commercial and industrial solvent due to its chemical properties. However, it can be particularly hazardous when ingested [1,2]. Methanol has a similar taste and odor to ethanol [3,4]; thus, it is sometimes used illicitly in place of ethanol during alcohol production. Alcoholic beverages may be contaminated with methanol. Both situations often arise from inadequate quality control procedures. In some cases, methanol is deliberately added to increase production volume and profit margins [2,5,6].

Methanol poisoning is associated with a high mortality rate globally and typically occurs following the ingestion of methanol-containing liquids or the consumption of illicit alcoholic beverages [7,8,9,10,11]. The toxic effects of methanol usually appear within 12 to 24 h after initial exposure due to its rapid absorption, metabolism, and large volume of distribution [12]. Early symptoms may resemble ethanol intoxication and include fatigue, nausea, and mild central nervous system (CNS) depression [13]. Consequently, affected individuals are often misdiagnosed or assumed to be intoxicated with ethanol, resulting in delays in appropriate medical intervention [4,14].

In the human body, methanol is first metabolized in the liver by alcohol dehydrogenase into formaldehyde, which is subsequently converted into formic acid and finally into carbon dioxide and water [15,16]. Although formaldehyde is highly toxic, it has a short half-life and does not accumulate in the body; therefore, significant concentrations are not typically detectable in tissues or body fluids following methanol ingestion [17]. Formic acid is the primary toxic metabolite of methanol and is largely responsible for the toxic effects and fatal outcomes observed in methanol poisoning [18,19].

The metabolism of methanol is influenced by various factors, including dose, genetic background, body weight, sex, and history of alcohol consumption. These factors also affect the severity of toxicity and the time interval between ingestion and death [2,20]. Hence, knowledge of the circumstances surrounding each case can provide critical insight into determining the cause of death. Although case series and literature reviews on methanol poisoning exist, there are relatively few comprehensive forensic evaluations. Here, 138 cases of methanol poisoning were retrospectively reviewed over a two-year period, including their autopsy findings, pathological results, and toxicological analyses. During forensic evaluations of the cause of death, discrepancies were observed between clinical signs and postmortem findings. The present study therefore provides a comprehensive evaluation of methanol poisoning cases in forensic medicine practice.

Despite the available literature, few studies have simultaneously investigated postmortem methanol and formic acid concentrations in multiple body fluids together with detailed organ-specific pathology, limiting the diagnostic interpretation of methanol-related fatalities. This study provides a comprehensive assessment of 138 autopsy-confirmed cases by integrating biochemical markers with macroscopic and microscopic pathological findings. By examining the relationship between toxic metabolite levels and characteristic injury patterns, this work offers novel diagnostic insights and contributes to a more accurate and refined postmortem evaluation framework for suspected methanol intoxication.

## 2. Materials and Methods

This study was approved by the Education and Scientific Research Commission of the Council of Forensic Medicine, Turkey (Approval No: 21589509/2025/372, dated 18 March 2025). A retrospective analysis was conducted on cases of methanol poisoning resulting in death, for which autopsies were performed at the Council of Forensic Medicine, Turkey, between January 2023 and March 2025. Autopsy reports were reviewed to collect data on autopsy findings, pathological examinations, and toxicological results. Demographic characteristics such as age, sex, and body mass index (BMI) were recorded along with pre-mortem symptoms, place of death, and cause of death. In addition, macroscopic autopsy findings and microscopic evaluations of the central nervous system, heart, lungs, liver, kidneys, and pancreas were included. Methanol and formic acid concentrations in postmortem blood and intraocular fluids were also documented. All cases included in the study represented individual methanol poisoning events; no mass casualty or outbreak-related cases were identified during the study period.

The diagnosis of methanol-related death was established through a comprehensive forensic evaluation integrating toxicological, pathological, and circumstantial findings. Accordingly, the diagnostic process did not rely solely on the presence of formic acid in postmortem biological samples. Cases were classified as methanol-related deaths when the following criteria were collectively satisfied: (i) detection of methanol in blood and/or vitreous humor at levels consistent with toxic exposure; (ii) documented clinical, circumstantial, or investigative evidence suggestive of methanol ingestion or exposure; (iii) presence of characteristic pathological findings commonly associated with methanol toxicity, particularly involving the central nervous system (e.g., putaminal discoloration or necrosis), considered supportive but not mandatory; and (iv) exclusion of alternative causes of death that could better explain the observed pathological and toxicological findings. In cases where formic acid levels were reported as zero, the diagnosis was supported by elevated methanol concentrations, compatible pathological findings, and contextual forensic information. It is also recognized that postmortem metabolism, delayed sampling, or advanced postmortem intervals may contribute to reduced or undetectable formic acid levels despite confirmed methanol intoxication.

Toxicological analyses of methanol and formic acid were performed by accredited forensic toxicology laboratories of the Council of Forensic Medicine as part of routine postmortem investigations. In this retrospective study, the authors evaluated toxicological results as reported in official laboratory records.

### Statistical Analysis

Statistical analyses were performed using IBM SPSS Statistics for Windows, Version 26.0 (IBM Corp., Armonk, NY, USA). Descriptive statistics were expressed as mean ± standard deviation (SD), minimum, maximum, frequency, and percentage, depending on the nature of each variable. The normality of distributions for continuous variables was evaluated using visual methods (histograms and Q–Q plots) and the Shapiro–Wilk test. Categorical variables were compared using Pearson’s chi-square test or Fisher’s exact test, as appropriate. For continuous variables, comparisons between independent groups were conducted using independent-samples *t*-tests when normal distribution assumptions were met. A two-tailed *p*-value < 0.05 was considered statistically significant, and where applicable, findings were also interpreted at more stringent significance thresholds of *p* < 0.01 and *p* < 0.001.

## 3. Results

A total of 138 autopsied cases of methanol poisoning were included in this study; 87% (n = 120) were male and 13% (n = 18) were female. The mean age was 49.73 ± 10.41 years (range: 22–74), and the mean body mass index (BMI) was 25.7 ± 5.6. When stratified by sex, the mean age was 45.78 ± 9.59 years for females and 50.33 ± 10.44 years for males, while the mean BMI values were 27.9 ± 7.75 and 25.37 ± 5.12, respectively. Seventy percent (n = 97) of the deceased were Turkish citizens. After alcohol consumption, 65.2% (n = 90) of individuals sought medical care at a hospital. The place of death was the hospital in 73.2% (n = 101) of cases, whereas 26.8% (n = 37) were found deceased at home.

Pre-mortem clinical symptoms included visual disturbances in 15.9% (n = 22), loss of consciousness in 30.4% (n = 42), nausea in 9.4% (n = 13), dizziness in 2.9% (n = 4), and dyspnea in 9.4% (n = 13). External examination revealed tattoos in 12.3% (n = 17) and self-inflicted scars or evidence of self-mutilation in 15.2% (n = 21). Table 1 summarizes the sociodemographic, clinical, and external examination findings.

The mean blood methanol concentration was 142.47 ± 139.20 mg/dL (range: 0–595), and the mean blood formic acid concentration was 258.62 ± 197.89 mg/dL (range: 0–618). In vitreous humor, the mean methanol concentration was 148.5 ± 145.53 mg/dL (range: 0–610), while the mean formic acid concentration was 287.04 ± 185.21 mg/dL (range: 0–597). Formic acid was detected in 89.1% (n = 123) of blood samples and 89.9% (n = 124) of vitreous humor samples.

Cases presenting with visual disturbances had statistically significantly lower mean methanol concentrations in both blood and vitreous humor compared with those without visual disturbances (*p* < 0.05). Similarly, mean formic acid levels were significantly lower in cases with visual disturbances than in those without (*p* < 0.05). Although no statistically significant differences were observed in methanol or formic acid concentrations between cases with and without loss of consciousness, mean levels tended to be lower in individuals without impaired consciousness (Table 2).

The mean weights of internal organs were as follows: brain, 1410 ± 153.09 g (range: 980–1831); heart, 414.68 ± 96.96 g (range: 228–843); combined lungs, 1433.21 ± 492.05 g (range: 547–3001); liver, 1894.71 ± 494.50 g (range: 1065–3354); combined kidneys, 354.07 ± 82.61 g (range: 181–587); and pancreas, 123.97 ± 53.98 g (range: 44–386) (Table 3).

Macroscopic examination revealed cerebral edema in 45.7% (n = 63) of cases and dark discoloration of the putamen in 31.9% (n = 44). Central nervous system hemorrhage was observed in 8.69% (n = 12). Cardiac ischemic changes were identified in 38.4% (n = 53). Hepatic steatosis was present in 61.6% (n = 85), and cirrhosis in 8.7% (n = 12) of liver samples. Renal cysts were detected in 15.9% (n = 22), while granular surface appearance was noted in 18.1% (n = 25).

When macroscopic findings were compared with methanol and formic acid concentrations in blood and vitreous humor, cases with hepatic steatosis had significantly higher mean methanol concentrations in both matrices (*p* < 0.05). Cases with cirrhosis showed elevated blood methanol and formic acid concentrations, as well as increased methanol levels in vitreous humor, although these differences were not statistically significant (*p* > 0.05). Further details are presented in Table 4.

Histopathological examination revealed cardiac ischemic changes in 38.4% (n = 53) of cases. Coronary artery narrowing was observed in 73.9% (n = 102). The most common pulmonary findings were edema (63.8%, n = 88), intra-alveolar hemorrhage (46.4%, n = 64), and pneumonia (33.3%, n = 46). Macrovesicular steatosis was the most frequent hepatic finding (61.6%, n = 85), followed by congestion (45.7%, n = 63). Acute pancreatitis was identified in 6.5% (n = 9) of pancreatic specimens. Detailed pathological findings are presented in Table 5.

Pathological cerebral hemorrhage was detected in 11.8% (n = 12) of cases who died in the hospital. There was a statistically significant association between hospital admission and the presence of pathological brain hemorrhage (*p* < 0.05). However, no significant correlation was found between the presence of formic acid in blood or vitreous humor and pathological brain hemorrhage (*p* > 0.05).

Ethanol was detected in the blood of 22.77% (n = 23) of hospitalized cases, and there was a statistically significant association between hospital admission and blood ethanol positivity (*p* < 0.05). Concomitant stimulant and/or narcotic substances were identified in 10.4% (n = 13) of cases. No statistically significant differences were observed in mean methanol or formic acid concentrations in blood or vitreous humor between cases with and without concomitant substance use (*p* > 0.05). Similarly, there were no significant differences in pathological organ findings between substance users and non-users (*p* > 0.05).

## 4. Discussion

Although numerous studies have examined the diagnosis and treatment of methanol poisoning, autopsy-based investigations remain relatively limited. Methanol intoxication causes significant morbidity and mortality, and elevated methanol concentrations in the body serve as a primary criterion for forensic diagnosis. However, in cases with lower methanol levels, interpretation often depends on variable postmortem findings. Fatal intoxications in forensic practice commonly result from ingestion of illicit alcoholic beverages or methanol-containing fluids. While nausea, abdominal pain, and visual disturbances are characteristic symptoms, severe intoxication may progress to delirium, coma, and death [2].

Methanol poisoning remains a critical issue in forensic medicine, particularly in cases of unexplained sudden death or fatalities associated with counterfeit alcohol. Postmortem toxicological analyses play a central role in establishing cause of death, and quantification of methanol and its metabolites in biological specimens, such as blood, vitreous humor, cerebrospinal fluid, and gastric contents, provides essential diagnostic information [21,22]. The predominance of male victims (87%) in our study is consistent with previous research. A nationwide retrospective study from Taiwan reported that 82.29% of methanol poisoning cases occurred in males [23], while an Iranian cohort reported 93% male fatalities [9].

This study provides one of the most comprehensive postmortem evaluations of methanol intoxication to date, integrating biochemical markers with detailed macroscopic and microscopic pathological findings in 138 autopsy-confirmed cases. Although substantial inter-individual variability was observed in methanol and formic acid concentrations, consistent pathological patterns affected the central nervous system, liver, lungs, and cardiovascular system. These findings align with previous reports highlighting formic acid–mediated metabolic acidosis as the primary mechanism of organ injury. By directly comparing metabolite levels in both blood and vitreous humor with organ-specific pathology, our study offers novel diagnostic insights that strengthen the interpretation of methanol-related fatalities.

The mean age of cases was 49.73 ± 10.41 years, indicating that methanol poisoning primarily affects middle-aged adults. This finding is comparable to prior literature, including a Norwegian study reporting a mean age of 53 years [24] and an Iranian report noting that 41% of cases occurred between 25 and 36 years old [9]. Sex-based BMI differences in our cohort may contribute to toxicokinetic variability; however, the influence of BMI on methanol toxicity remains unclear, warranting further investigation.

A total of 65.2% of individuals presented to a healthcare facility before death, whereas 34.8% died without receiving medical attention. This reflects delayed recognition of symptoms, which typically manifest 12–24 h after exposure and may initially resemble ethanol intoxication [3]. Early diagnosis and prompt treatment are crucial, as delayed intervention is associated with higher mortality.

In our study, 73.2% of deaths occurred in the hospital, while 26.8% occurred at home. Deaths outside medical settings likely reflect uncontrolled use, unrecognized toxicity, or barriers to accessing care. Similar studies have shown that timely clinical intervention significantly reduces mortality [24,25]. Despite hospital presentation, mortality remained high in our cohort, underscoring the severity of methanol toxicity.

Visual impairment was a notable premortem symptom (15.9%), consistent with its recognized role as a hallmark feature of methanol poisoning. Formic acid has been shown to inhibit mitochondrial cytochrome oxidase, disrupting oxidative metabolism and causing irreversible retinal and optic nerve injury [26]. Early visual symptoms are important prognostic indicators, and timely antidotal therapy significantly decreases the risk of permanent vision loss [3,24].

Interestingly, both methanol and formic acid concentrations were significantly lower in cases presenting with visual disturbances compared with those without such symptoms. Although counterintuitive, clinical and experimental evidence indicates that formic acid can cause optic nerve injury even at moderate exposure levels, and visual toxicity cannot be reliably predicted solely by systemic metabolite concentrations [3,26,27]. Individuals who develop early visual symptoms may seek medical care sooner, resulting in biological sampling prior to peak metabolite accumulation. In addition, susceptibility to optic nerve toxicity varies markedly between individuals, and lower formic acid concentrations may still produce significant visual impairment in vulnerable patients. Recent evidence highlights substantial inter-individual variability in visual toxicity thresholds, with visual injury severity not directly correlating with formic acid concentration alone [28]. These findings emphasize the importance of integrating clinical symptoms with metabolic and pathological data rather than relying solely on quantitative toxicology results.

Tattoos and self-mutilation scars, present in 12.3% and 15.2% of cases, respectively, may reflect underlying psychiatric vulnerability or intentional ingestion in certain individuals. Supporting this interpretation, Kordrostami et al. (2017) reported that a portion of methanol poisoning cases result from intentional rather than accidental consumption [29]. A Norwegian outbreak investigation similarly documented deliberate ingestion in approximately 28% of cases [24]. Another study found that individuals with severe psychiatric symptoms or higher formic acid production were more likely to ingest methanol intentionally [27]. These findings highlight the importance of psychiatric evaluation and targeted preventive strategies in at-risk groups.

In our cohort, mean blood methanol and formic acid concentrations were 142.47 ± 139.20 mg/dL and 258.62 ± 197.89 mg/dL, respectively, with corresponding vitreous humor concentrations of 148.5 ± 145.53 mg/dL and 287.04 ± 185.21 mg/dL. These results demonstrate rapid conversion of methanol to formic acid and accumulation of both metabolites in systemic circulation and ocular compartments. Elevated intraocular formic acid levels may contribute to the pronounced ocular toxicity associated with methanol poisoning. Barceloux (2002) reported that inhibition of mitochondrial cytochrome oxidase by formic acid disrupts cellular respiration, leading to irreversible injury in metabolically active tissues such as the retina and optic nerve [3]. In vitro studies further show that formic acid induces ATP depletion and cytotoxicity in photoreceptor and retinal pigment epithelial cells, reinforcing its pathogenic role.

Formic acid was detected in both blood and intraocular fluid in more than 89% of cases, indicating that the toxic effects of methanol persist systemically as well as in ocular compartments. Supporting this, Ghorbani et al. (2018) emphasized the importance of measuring both methanol and formic acid in postmortem specimens and demonstrated that high concentrations of formic acid can be reliably quantified in intraocular fluid, providing valuable information on the degree of toxicity and its contribution to the cause of death [30]. Likewise, Wallage and Watterson (2008) reported intraocular and blood formic acid concentrations ranging between 64 and 110 mg/dL in fatal methanol cases, confirming that formic acid serves as a critical biomarker for determining toxic exposure [31]. A clinical study by Hovda et al. (2005) further demonstrated that elevated formic acid levels correlate with severe metabolic acidosis and optic nerve injury, underscoring its central role in methanol toxicity [24].

In cases presenting with visual impairment, mean blood and intraocular fluid methanol and formic acid levels were significantly lower than in cases without visual impairment (*p* < 0.05), a finding that appears contradictory to the classical understanding of methanol toxicity. Jacobsen and McMartin (1986) described a dose-dependent association between formic acid accumulation and optic nerve injury, with higher levels increasing the risk of visual loss [26]. However, our results suggest a more complex relationship between toxic metabolite levels and clinical manifestations. Factors such as the timing of exposure, duration of poisoning, redistribution of formic acid into tissues, and postmortem sampling intervals may influence measured concentrations. For example, many of the individuals in this study died after hospital admission, meaning postmortem formic acid levels may not reflect peak concentrations present at symptom onset. Supporting this, Paasma et al. (2007) showed that initial blood methanol and formic acid levels do not always correlate directly with the severity of visual disturbances [32]. Consistent with previous literature, no significant differences in metabolite levels were observed between cases with and without loss of consciousness, further indicating that the neurological manifestations of methanol poisoning cannot be explained solely by biochemical parameters and are likely influenced by individual susceptibility and clinical circumstances [24,26].

Postmortem internal examinations included detailed assessments of organ weights as well as macroscopic and histopathological findings of the brain, heart, lungs, liver, kidneys, and pancreas. The mean brain (1410 ± 153.09 g), heart (414.68 ± 96.96 g), and combined lung weights (1433.21 ± 492.05 g) were consistent with the edema and congestion frequently reported in methanol-related fatalities [27,32]. Cerebral and pulmonary edema are systemic reflections of methanol intoxication and often accompany severe metabolic acidosis. These organ weight abnormalities, together with the macroscopic findings, provide important insights into the organ-level consequences of methanol poisoning and the characteristic pathological changes observed at autopsy [32,33].

Central nervous system edema was identified in 45.7% of cases, while dark discoloration of the putamen was observed in 31.9%. These findings highlight methanol’s well-established neurotoxic profile, as formic acid exerts its most detrimental effects on metabolically active regions of the brain, particularly the basal ganglia. The putamen is especially vulnerable due to its high metabolic demand and limited ability to buffer formic acid–induced mitochondrial inhibition. Previous MRI and pathological studies have shown that putaminal lesions often appear as dark discolorations, frequently associated with necrosis or hemosiderin deposition in methanol poisoning [26,27]. The presence of pathological intracranial hemorrhage in a subset of cases further reflects the severity of metabolic acidosis and microvascular injury associated with advanced toxicity.

Brain hemorrhage is a rare but serious complication of methanol poisoning and appears to occur more frequently in individuals presenting with severe clinical deterioration requiring intensive care support. Although basal ganglia injury, cerebral edema, and occasional intracranial hemorrhage have been described in the literature, definitive data on the prevalence and mechanisms of methanol-related brain hemorrhage remain limited [26,34]. The hemorrhagic changes observed in our study likely reflect advanced metabolic acidosis, endothelial dysfunction, and microvascular injury, all of which contribute to the progression of severe neurotoxicity.

Cardiac pathology was also prominent: ischemic changes were identified in 38.4% of cases. This finding suggests that the systemic toxicity of methanol extends beyond the central nervous system and causes significant structural injury in cardiac tissue. Formic acid disrupts mitochondrial oxidative phosphorylation, resulting in metabolic acidosis, cellular hypoxia, and impaired energy production-mechanisms that can precipitate myocardial ischemia [35]. Additionally, the presence of coronary artery narrowing due to chronic comorbidities may further exacerbate susceptibility to cardiac injury following methanol exposure [36]. Together, these findings highlight that methanol toxicity is a multisystem condition with the potential to cause life-threatening damage in both neural and cardiac tissues.

Liver steatosis was identified in 61.6% of cases and cirrhosis in 8.7%, suggesting that methanol toxicity disrupts hepatic lipid metabolism and may exacerbate pre-existing liver disease rather than directly causing chronic pathology [37]. Macrovesicular steatosis demonstrated a significant correlation with methanol concentrations in both blood and vitreous humor, indicating that methanol’s hepatotoxic effects and the metabolic disturbances associated with acute poisoning contribute to lipid accumulation in hepatocytes. Methanol metabolism generates formic acid, which induces oxidative stress, mitochondrial dysfunction, and inflammatory responses—mechanisms that are known to promote steatosis. Metabolic acidosis and impaired energy homeostasis during severe intoxication may further aggravate hepatic lipid dysregulation. Consistent with our findings, previous forensic studies have frequently reported micro- and macrovesicular steatosis, focal hepatocyte necrosis, cholestasis, and hydropic degeneration in fatal methanol intoxications, with more pronounced changes observed at higher methanol concentrations [38,39]. An Iranian histopathological study similarly demonstrated widespread steatosis and hepatocyte injury in fatal cases, particularly when blood methanol levels exceeded 127 mg/dL [40].

Renal pathology was also notable: kidney cysts were present in 15.9% of cases and granular cortical appearance in 18.1%, supporting the notion that methanol induces structural alterations in renal tissue and may impair renal filtration capacity [35]. These renal findings likely reflect systemic metabolic acidosis, toxic metabolite accumulation, and oxidative stress, all of which contribute to multisystem involvement in methanol poisoning. Taken together, the hepatic and renal abnormalities observed in this study underscore that methanol intoxication is not solely a neurotoxic event but a multisystemic pathological process that significantly increases morbidity and mortality.

Pulmonary edema was observed in 63.8% of cases, intra-alveolar hemorrhage in 46.4%, and pulmonary infection in 33.3%, indicating that both the direct toxic effects of methanol and secondary clinical complications contribute to severe pulmonary pathology. Autopsy and histopathological examinations consistently demonstrate that the lungs are among the organs most profoundly affected in methanol poisoning, reflecting the combined effects of metabolic acidosis, hypoxia, and circulatory instability. An autopsy-based investigation from India similarly reported widespread cerebral and pulmonary edema, as well as congestive changes in the lungs, during large methanol outbreaks in the Barabanki and Sitapur regions—findings often associated with sepsis-related pulmonary edema [33]. The high prevalence of pulmonary edema in our study is therefore consistent with previously published outbreak data.

Formic acid, the principal toxic metabolite of methanol, inhibits mitochondrial oxidative pathways and compromises cellular energy production, leading to systemic acidosis and tissue hypoxia. These mechanisms increase pulmonary capillary permeability, a key driver of pulmonary edema. In addition, severe neurological deterioration may necessitate intubation and mechanical ventilation, which increases the risk of aspiration and secondary pulmonary infections. Such clinical circumstances may also predispose affected individuals to intra-alveolar hemorrhage, further exacerbating respiratory compromise [41].

Acute pancreatitis was identified in 6.5% of cases, indicating that the systemic toxicity of methanol may also extend to pancreatic tissue. Although uncommon, pancreatic injury has been documented in the literature. Hantson and Mahieu (2000) reported pancreatic involvement in 11 of 22 patients with methanol poisoning, including cases of moderate to severe acute pancreatitis and one instance of necrotizing pancreatitis resulting in death [42]. In addition, contemporary reviews of methanol poisoning describe pancreatic involvement as part of the broader spectrum of formic acid–mediated multi-organ toxicity [35]. Pancreatic hemorrhage has also been reported as a fatal complication in autopsy-based investigations. The mechanisms underlying pancreatic injury may involve direct toxic effects of methanol and formic acid or oxidative stress–mediated free radical damage. While ethanol administration and chronic alcohol use may exacerbate pancreatic inflammation, the presence of pancreatitis before initiation of treatment in some cases suggests that methanol’s systemic toxicity alone may be sufficient to trigger pancreatic injury. Although relatively infrequent, the 6.5% prevalence observed in our study underscores its clinical significance, and monitoring pancreatic enzymes may be valuable during both diagnostic evaluation and treatment follow-up.

Pathological brain hemorrhage was identified in 11.8% of individuals who died in the hospital, with a statistically significant association between hospital admission and the presence of hemorrhage (*p* < 0.05). This finding suggests that patients who reach medical care tend to present with more severe clinical deterioration, making them more susceptible to advanced neurovascular complications. The presence of brain hemorrhage highlights the severe neurological consequences of methanol poisoning and reinforces the importance of rapid hospital-based intervention in preventing further central nervous system injury.

Formic acid, the principal toxic metabolite of methanol, causes profound neurotoxicity and can lead to hemorrhagic lesions in metabolically sensitive brain regions such as the basal ganglia, as documented in recent clinical reports of intracranial hemorrhage in methanol poisoning [43,44]. More recent case reports have further expanded the spectrum of neurological complications. Li et al. (2022) described bilateral diffuse cerebral hemorrhage in a patient with acute methanol intoxication [45], while Yolay et al. (2023) documented a detailed clinical course of intracranial hemorrhage complicating methanol poisoning [46]. These observations confirm that although brain hemorrhage is uncommon, it represents a serious manifestation of severe metabolic acidosis, endothelial injury, and microvascular compromise.

The increased incidence of brain hemorrhage among hospitalized cases in our study underscores the association between severe clinical deterioration and neurovascular injury. Notably, no significant correlation was observed between blood or vitreous humor formic acid concentrations and the presence of brain hemorrhage, consistent with previous findings suggesting that toxic metabolite levels alone do not fully predict neurological complications [27,31]. This indicates that individual susceptibility, duration of exposure, tissue accumulation, and comorbid conditions play critical roles in determining the extent of CNS injury. These insights emphasize the importance of rapid clinical evaluation and timely treatment to mitigate the progression of neurological damage.

Ethanol was detected in 22.77% of hospitalized individuals, a statistically significant finding that carries important clinical and forensic implications. The presence of ethanol may reflect either concomitant ingestion or therapeutic administration during treatment. While ethanol competitively inhibits alcohol dehydrogenase and reduces the formation of toxic metabolites, concurrent intake may complicate the metabolic course of poisoning and obscure symptom presentation. Previous studies have highlighted ethanol’s therapeutic benefits but have also noted that simultaneous consumption can alter clinical outcomes and complicate postmortem toxicological interpretation [47,48]. Therefore, ethanol detection must be evaluated cautiously in both clinical management and forensic assessment.

Polydrug exposure involving narcotics or stimulants was identified in 10.4% of cases. Such substances can potentiate methanol-induced metabolic disturbances and increase risks of neurotoxicity and cardiotoxicity. Stimulants such as cocaine or amphetamines may exacerbate metabolic acidosis and oxygen demand, while narcotics may worsen respiratory suppression-both significantly complicating the clinical trajectory. As shown in previous toxicological studies, polydrug use requires heightened clinical vigilance and comprehensive toxicological screening during both diagnosis and postmortem evaluation [49].

This study has several limitations that should be acknowledged. Information regarding hospital admission prior to death, duration of hospital stay, and exact postmortem interval (PMI) was not consistently available in the retrospective case files and therefore could not be systematically included in the analysis. As a retrospective autopsy-based investigation, the study relies on available medical records and postmortem documentation, which may lack detailed clinical timelines or treatment histories. Additionally, postmortem metabolite levels may not fully reflect concentrations at the time of symptom onset, particularly in individuals who survived for extended periods before death. In many fatal methanol intoxications, the intent of ingestion cannot be reliably determined. In routine forensic practice, individuals are often unaware that the consumed alcoholic beverage contains methanol, particularly in cases involving illicit or counterfeit alcohol. Consequently, distinguishing between accidental and intentional exposure is frequently not feasible based solely on postmortem findings or retrospective case records. This uncertainty limits definitive classification of the manner of death and underscores the need for cautious interpretation in forensic evaluations.

## 5. Conclusions

Methanol poisoning remains a significant global public health problem, leading to substantial morbidity and mortality. While its clinical manifestations and treatment strategies have been extensively studied, autopsy-based investigations are comparatively limited. In forensic practice, markedly elevated methanol concentrations are central to establishing the diagnosis; however, in cases with lower levels or prolonged survival, interpretation must rely on variable postmortem findings and integrated pathological assessment. Fatal intoxications frequently result from the consumption of illicit alcoholic beverages or other methanol-containing products, and both clinical presentations and postmortem findings may vary considerably among individuals.

This retrospective study provides a comprehensive evaluation of demographic, toxicological, macroscopic, and histopathological characteristics in cases of fatal methanol poisoning. By integrating autopsy findings with quantitative measurements of methanol and formic acid in both blood and vitreous humor, the study enhances diagnostic precision and offers valuable insight into organ-specific injury patterns, particularly within the central nervous system, liver, lungs, and pancreas. The observed associations between metabolite levels and pathological findings underscore the importance of a multidimensional forensic approach.

A key contribution of this study is the demonstration of how combined interpretation of biochemical markers and organ-specific pathology can clarify diagnostic uncertainty in cases where methanol concentrations alone are insufficient. Notably, the finding of lower methanol and formic acid levels in individuals with visual impairment highlights the need to consider toxicokinetic variability and tissue-level accumulation rather than relying solely on quantitative thresholds. This integrative approach provides novel diagnostic insight that can improve forensic decision-making in complex cases.

## Figures and Tables

**Table 1:** Sociodemographic and Clinical Characteristics.

		n	%
Sex	Male	120	87.0
	Female	18	13.0
Nationality	Citizen	97	70.3
	Non-Citizen	39	28.3
	Unknown	2	1.4
Event Type	Hospital Admission	90	65.2
	Found Dead	37	26.8
	Found Unconscious	11	8.0
Place of Death	Hospital	101	73.2
	Home	37	26.8
Tattoo	Present	17	12.3
Self-mutilation	Present	21	15.2
Visual Impairment	Present	22	15.9
	Absent	65	47.1
	N/A	51	37.0
Loss of Consciousness	Present	42	30.4
	Absent	45	32.6
	N/A	51	37.0
Nausea	Present	13	9.4
	Absent	73	52.9
	N/A	52	37.7
Dizziness	Present	4	2.9
	Absent	81	58.7
	N/A	53	38.4
Shortness of Breath	Present	13	9.4
	Absent	73	52.9
	N/A	52	37.7

N/A: Not Available.

**Table 2:** Comparison of Blood and Vitreous Methanol and Formic Acid Levels by Clinical Findings.

Clinical Finding	Blood Methanol Level	Blood Formic Acid Level	Vitreous Methanol Level	Vitreous Formic Acid Level
Visual Impairment				
Present	51.32 ± 55.00 **	117.89 ± 157.42 *	58.91 ± 60.24 **	195.00 ± 193.21
Absent	116.58 ± 116.36 **	239.36 ± 186.62 *	118.09 ± 123.06 **	280.74 ± 193.84
Loss of Consciousness				
Present	92.90 ± 114.61	190.88 ± 183.53	95.95 ± 118.72	241.34 ± 195.77
Absent	106.98 ± 102.31	221.03 ± 190.30	110.00 ± 109.15	277.16 ± 197.30
Cerebral Edema				
Present	92.78 ± 108.53	187.59 ± 176.66	87.63 ± 95.34	261.36 ± 193.04
Absent	108.45 ± 108.02	229.68 ± 198.47	120.45 ± 129.31	255.78 ± 203.00
Pathological Hemorrhage				
Present	90.75 ± 118.62 *	145.35 ± 203.52 **	93.32 ± 131.22 *	146.18 ± 188.49 ***
Absent	155.64 ± 141.45 *	298.71 ± 180.99 **	162.68 ± 146.20 *	335.47 ± 158.52 ***

All values are presented as mean ± standard deviation (SD). Methanol levels are expressed in mg/dL, and formic acid levels in mg/dL. Independent samples *t*-tests were used for group comparisons. Values marked with * indicate *p* < 0.05; ** indicate *p* < 0.01; *** indicate *p* < 0.001.

**Table 3:** Descriptive Statistics of Organ Weights.

Organ	Mean (g)	Standard Deviation	Minimum	Maximum
Brain	1410.14	153.97	980.00	1831.00
Heart	414.68	96.93	228.00	843.00
Lungs	1433.21	492.05	547.00	3001.00
Liver	1894.71	494.50	1065.00	3354.00
Kidney	354.07	82.61	181.00	587.00
Pancreas	123.78	53.98	44.00	386.00

All values are presented in grams (g) as mean, standard deviation, minimum, and maximum. Lung and kidney weights represent the total of both organs.

**Table 4:** Methanol and Formic Acid Levels by Pathological Findings of the Organs.

Autopsy Finding	Blood Methanol Level	Blood Formic Acid Level	Vitreous Methanol Level	Vitreous Formic Acid Level
Putamen/Basal Ganglia Reddish Color Changed				
Present	128.98 ± 120.43	237.97 ± 175.80	144.66 ± 136.67	286.52 ± 156.99
Absent	148.79 ± 147.36	271.02 ± 210.63	150.32 ± 150.23	287.38 ± 202.26
Hepatic Steatosis				
Present	163.53 ± 150.36 *	270.27 ± 204.58	168.96 ± 157.83 *	270.80 ± 180.84
Absent	108.70 ± 112.46 *	244.00 ± 190.79	116.08 ± 117.84 *	308.57 ± 191.20
Liver Cirrhosis				
Present	173.83 ± 173.85	279.14 ± 229.20	169.83 ± 184.69	235.57 ± 194.36
Absent	139.48 ± 135.93	256.85 ± 196.50	146.46 ± 141.97	291.61 ± 184.98
Acute Pancreatitis				
Present	80.22 ± 139.99	165.88 ± 247.43	87.89 ± 151.59	182.63 ± 240.29
Absent	146.81 ± 138.65	267.90 ± 191.70	152.77 ± 144.76	297.76 ± 177.12
Coronary Artery Disease				
Present	135.28 ± 132.88	250.85 ± 206.07	139.31 ± 135.47	275.10 ± 193.12
Absent	162.83 ± 155.96	276.19 ± 180.53	174.31 ± 170.11	314.62 ± 165.77

All values are presented as mean ± standard deviation (SD). Methanol levels are expressed in mg/dL, and formic acid levels in mg/dL. Independent samples *t*-tests were used for group comparisons. Values marked with * indicate *p* < 0.05.

**Table 5:** Histopathological Findings of the Organs.

Organ	Histopathological Finding	n	%
Heart	Ischemic Changes	53	38.4%
	Coronary Artery Occlusion	102	73.9%
	Cardiac Hypertrophy	26	18.8%
	Myocardial Fibrosis	72	52.2%
Lung	Pulmonary Edema	88	63.8%
	Intra-alveolar Hemorrhage	64	46.4%
	Pulmonary Infection	46	33.3%
	Pulmonary Embolism	1	0.7%
	Pulmonary Thrombus	15	10.9%
Liver	Cirrhosis	12	8.7%
	Steatosis	85	61.6%
	Portal Inflammation	32	23.2%
	Congestion	63	45.7%
Pancreas	Acute Pancreatitis	9	6.5%
	Chronic Pancreatitis	2	1.4%
Kidney	Renal Cyst	22	15.9%
	Granular Surface	25	18.1%

## Data Availability

The datasets used and/or analyzed during the current study are available from the corresponding author on reasonable request.
